# Learning Methods During School Closure and Its Correlation With Anxiety and Health Behavior of Thai Students

**DOI:** 10.3389/fped.2022.815148

**Published:** 2022-03-28

**Authors:** Dyah Anantalia Widyastari, Sarocha Kesaro, Niramon Rasri, Pairoj Saonuam, Piyawat Katewongsa

**Affiliations:** ^1^Institute for Population and Social Research, Mahidol University, Nakhon Pathom, Thailand; ^2^Thailand Physical Activity Knowledge Development Centre (TPAK), Institute for Population and Social Research, Mahidol University, Nakhon Pathom, Thailand; ^3^Thai Health Promotion Foundation, Bangkok, Thailand

**Keywords:** learning methods, school closure, COVID-19 pandemic, healthy behavior, anxiety, children

## Abstract

**Background:**

The sequential waves of epidemic spread of COVID-19 in Thailand have caused periodic closures of schools, and exposed students to different learning methods that require multiple adjustment strategies. This study aimed to examine how different learning methods may correlate with anxiety and health behavior (e.g., physical activity, active play, screen time, sleep) of primary and secondary school students in Thailand.

**Methods:**

Thailand Report Card (TRC) Data (2021) was employed. The sample of the TRC was drawn by multi-stages random sampling stratified by region, district, urban/rural, school size, sex, and age to ensure national representativeness. A total of 6,078 Thai primary (64%) and secondary (36%) school students were included in the analysis.

**Results:**

About two-thirds (66.4%) of the sample experienced a shift from traditional classroom to fully online learning, 6.9% experienced partial online instruction, 23.6% received handouts or written assignments, and 3.1% resumed traditional classroom learning. Compared to fully online learning, students who experienced traditional classroom (onsite) teaching were 37.8% less likely to report moderate-to severe anxiety (OR 0.6; *p*-value 0.021). There was no significant correlation between school closure-induced anxiety with overall physical activity (PA) and active play, but anxiety was significantly associated with screen time and sleep duration. Receiving handouts/written assignments only as the learning method was significantly correlated with PA, but two methods (handouts and onsite/traditional classroom) was significantly correlated with active play. Students who experienced classroom learning were also more likely to comply with recommended durations of screen time and sleep.

**Conclusion:**

Although online learning was probably the most convenient choice during COVID-19 containment measures in Thailand, this method did not provide sufficient opportunity for PA and play. Online learning also encouraged an excessive use of screen media, and disrupted sleeping patterns. Online learning also pressured Thai students to make various adjustments in their daily routines that may have further aggravated anxiety.

## Introduction

Since the emergence of the COVID-19 pandemic in late 2019, children and youth have been adversely affected – if not directly by illness – at least indirectly by significant disruptions to their daily life. In 2020, an estimated 1.6 billion students in 190 countries were unable to attend school as usual (1). Sequential waves of spread of COVID-19 have caused periodic closures of schools, and required students to experience a range of teaching methods that require multiple adjustment strategies. Home learning indeed offers many benefits for students with its flexibility (2, 3), cost and time saving from eliminating the daily commute to/from school (4), and extra time to review lesson materials as frequently as needed (3). However, home learning has unfortunately widened educational disparities (5). Remote learning requires the collaborative involvement of children, parents/guardians, teachers, and the schools to ensure the children actively participate in the scheduled program of instruction (6, 7).

Many studies have reported an alarming level of anxiety and stress among youth caused by the compulsory use of alternative learning methods following school closures (6, 8, 9). Students are confined to their home and required to engage in repetitive daily routines that may result in boredom, loneliness, and stress (6, 10). Home learning may also induce anxiety for children who are slow to adapt to the remote learning methods, or those who are accustomed to peer support at school (6). In addition to anxiety, increased sleep duration and changes in waking patterns for school-age youth was also reported during periods of school closure (11). Youth also accumulated 2–3 h higher screen time than during the pre-pandemic period (12–14). The physical activity (PA) was also significantly declined during periods of lockdown, and a further decline was observed when the duration of school closure was prolonged (6, 14).

In Thailand, the first school closure to contain the spread of COVID-19 was ordered by the Ministry of Education in early 2020, followed by the postponement of the beginning of the academic year from May to July in 2020, and from June 1st to 14th in 2021 (15, 16). Since then, most Thai students have experienced different types of adjustments to substitute classroom learning. For primary school students (grades 1–6), one-way communication (e.g., homework assignments) was mostly used, which greatly relied on parents/guardians to guide or oversee their child’s compliance. For secondary school students, Distance Learning Television (DLTV) and/or online learning methods were introduced (17).

While the benefits and disadvantages of home/online learning have been documented even before the pandemic, little is known how the changes in the learning methods during school closure may have an effect to Thai children’s anxiety and their health behaviors. With various learning methods being implemented during school closure and changing over time, students might experience frustration and stress from the fluctuating learning environment and policy. Thus, this study aimed to examine how different learning methods may correlate with anxiety and health behavior (e.g., PA, active play, screen time, and sleep) of primary and secondary school students in Thailand. The hypothesis of this study stands on the argument that online learning may not be the best method for a child’s educational development but, given the unpredictable threat of new outbreaks of COVID-19, it might be the preferable option. On the other hand, written assignments or non-online home learning methods provide flexibility in time management, and with less potential SES constraints that come with online learning.

## Materials and Methods

### Data, Population, and Sample

This study employed data from the 2021 Thailand Report Card (TRC), a nationally representative survey of Thai students aged 5–17 years. With school closures due to outbreaks of COVID-19 in many provinces around the country, and considering that 85% of the Thai population had Internet access (18), the survey designated Thai children and youth whose parents, family or themselves have the access to the Internet as the population of the study. The sample of the TRC was derived by multi-stage random sampling. First, two provinces were selected randomly from the four geographic regions of the country and Bangkok. Then, one urban and one rural district from each province was selected. For each selected district, schools were sampled with probability proportional to size (i.e., small, medium, and large). The inclusion criteria for participants are as follows: (i) aged 5–17 years, (ii) they or their parents/family have the access to the Internet, (iii) able to communicate in Thai, and (iv) consented to participate in the survey. The sample size was determined by using this following formula:


$$n=\frac{N\,Z_{1-\alpha /2}^{2}\,\sigma^{2}/{\bar{X}}^{2}}{N\,\varepsilon^{2}+Z_{1-\alpha /2}^{2}\,\sigma^{2}/{\bar{X}}^{2}}$$

where, *n* denotes sample size, *N* is the population of students (5,708,597), *$\bar{X}$* is the average minutes of MVPA daily of each stratum, σ is the standard deviation of the average minutes of MVPA daily of each stratum, *Z* is the confidence interval (1.96), and the precision *(ε)* was defined at 0.20 with the margin of error *(α)* at 0.05. While at least 5,940 students required to be representative of the population following the abovementioned formula, this study collected a complete data of 6,078 students (64% primary and 36% secondary) for the analysis. The sample characteristics is shown in Table 1.

**TABLE 1:** Participant characteristics.

	*N* = 6078	%
**Sex**		
Female	3076	50.6
Male	3002	49.4
**Education level**		
Lower primary	1983	32.6
Upper primary	1935	31.9
Lower secondary	1332	21.9
Upper secondary	828	13.6
**Place of residence**		
Urban	3975	65.4
Rural	2103	34.6
**Region of residence**		
North	1214	20.0
Northeast	1212	19.9
Central	1837	30.2
South	1204	19.8
Bangkok	611	10.1

An “on screen face-to-face interview” was used for data collection. In this application, the interviewer and respondent were in a real-time, face-to-face interaction using a screen medium (e.g., smartphone, tablet, and laptop) connected to the Internet. This method was considered the most effective considering the following: (i) travel restrictions had made it impossible to travel between regions during the scheduled period of data collection, (ii) a self-administered online questionnaire was considered inappropriate for students in this age group, and (iii) the method was assumed to yield a better response rate and data quality compared to a self-administered online survey. LINE video call, Facebook messenger video, or Zoom were used as the medium, based on respondent preference. Interviews were conducted by trained personnel using the LimeSurvey web application open-source version 5.1.17 build 211025 licensed to the Thailand Physical Activity Knowledge Development Centre.

### Assessment of Variables

“Learning method” was defined as the type of formal education during periods of school closure, grouped into the following: (1) fully online, any platform (e.g., online classroom, flipped classroom); (2) partially online, includes a combination of online and other methods (e.g., DLTV and online); (3) handouts and/or written assignments; and (4) onsite (traditional, in-person classroom learning).

“Anxiety” in this study refers to school closure-related anxiety. It was measured using a 5-point Likert scale ranging from “very mild” to “very severe,” in response to a question on the level of stress the respondent felt due to the school closure. To help prompt response, a flashcard containing images of five emotions was shown. For the analysis, response was grouped into (1) very mild-to-mild; and (2) moderate-to-severe anxiety.

Duration of PA, active play, screen time, and sleep was calculated by using a 24-h activity log. Respondents were asked to recall their hourly activities starting at the time they woke up until they went to bed, and response was categorized as PA, active play, screen time, or sleep. The students were also requested to describe how much likelihood their activities have caused a large increase in breathing and/or heart rate or have caused them feeling tired as a measure of PA intensity (light, moderate, or vigorous). The types of activities were classified according to the Thai Physical Activity Guideline (TPAG) in defining PA level (19). For the analysis, overall PA was categorized into the following: (1) meets the recommended guideline of 60 min MVPA daily; and (2) does not meet the recommended PA guideline.

Considering the purpose and unstructured nature of play for children, active play was defined as a form of PA that usually involves gross motor movement, random activity, freely chosen, and occurring outdoors (20). The duration of outdoor active play at any intensity was calculated. Global Matrix 3.0 (21) benchmark on active play was used in categorizing active play into the following: (1) meets the active play guidelines (being outdoors for more than 2 h per day); and (2) does not meet the active play guideline.

For each activity that involved screen media, participants were asked to estimate the duration and purpose of screen-related activities per day. This study only included recreational screen activities, and grouped response based on the Canadian sedentary behavior guidelines for students (22): (1) meets the screen time guideline (less than 2 h recreational screen time per day); and (2) does not meet screen time guidelines (2 or more hours recreational screen time per day).

Guidelines of the National Sleep Foundation (23) were used to classify recommended sleep duration for respondents aged 6–13 years (9–11 h), and aged 14–17 years (8–10 h), and response was coded as (1) meets sleep guidelines; and (2) does not meet sleep guideline.

### Covariates

Socio-demographic data such as sex, grade, family type, area of residence, and region of residence were analyzed to provide the context of response. Participants were differentiated by their sexes whether they are (1) males, or (2) females, and grouped their education into (1) lower primary, (2) upper primary, (3) lower secondary, and (4) upper secondary. Residential characteristics grouped the respondents into (1) urban or (2) rural, and by its regions (1) Northern, (2) Northeast, (3) Central (4) Southern, or (5) Bangkok Metropolis. This study also analyzed family/peer support, and whether the respondent had a debilitating chronic disease that may limit PA.

### Data Analysis

ANOVA (*F*-test) was employed to test the mean difference of anxiety among four learning methods, whereas Chi-square was used to test for an association between anxiety and behavior (PA, screen time, and sleep). Multivariate analysis with binary logistic regression was used to (1) determine the correlation between learning methods and anxiety, and (2) establish the correlation between anxiety and PA, active play, screen time and sleep. All covariates were included in the model, with a significance level of *p* = 0.05 or less to determine whether a variable had an effect on the designated behavior or its outcome. IBM SPSS version 25 (IBM Corp., Armonk, NY, United States) licensed to Mahidol University was used for most of the data analysis. The AMOS program was used to run a path analysis as an additional examination of the direct and indirect association between variables.

### Ethical Considerations

The protocol for the Thailand Report Card Survey (2021) received ethical approval from the Institutional Review Board of the Institute for Population and Social Research, Mahidol University, COA. No. 2021/05-115. Since this study involved minors, a scan/image of written informed consent was obtained from a parent/guardian of the respondent prior to the interview. The researchers clearly explained the purpose of the study and right to withdraw at any time for any reason. A parent/guardian was also allowed to be present during the interview with their child.

## Results

### Learning Methods During School Closures

**FIGURE 1:**
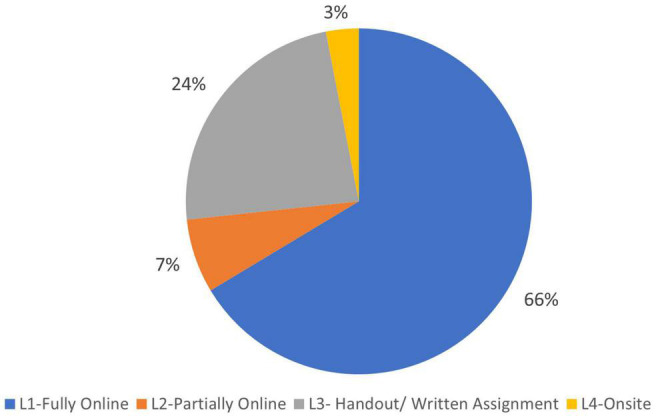
Percentage of Thai students by learning method during the COVID-19 epidemic.

Thai school closures to contain the spread of COVID-19 were first ordered by the Ministry of Education in April 2020. Accordingly, most Thai students shifted their in-class coursework to home learning. To allow time for schools to adjust to remote learning, the Ministry of Education postponed the new academic semester from May to July 2020 (17). Of 6,078 sample that can be generalized to Thai population, this study found that 66.4% of Thai students switched to fully online learning, whereas 6.9% used partially online methods (Figure 1).

Fully online learning was only possible for students with continuous, adequate access to the Internet and supporting facilities. Structured online classes were scheduled from Monday to Friday, for an average of 5 h for primary school grades, and 6 h for secondary school grades. Each subject consisted of a one-hour session, and the teacher delivered the content through video conferencing (e.g., Zoom), with 10–20 min off-screen break for each session. Apart from the structured online classroom instruction provided by the schools, any student throughout Thailand could access the online learning platform developed by the Office of Private Education Commission (OPEC) (24). Assignments were also provided to enhance student understanding on the given subjects. For younger students, parents could complement their child’s online learning with interactive activities such as drawing, book reading, building blocks, or conversation for practicing language.

Partially online methods aimed to accommodate those students without adequate access to the Internet, and that was implemented through free distance-learning TV (DLTV) and other platforms. DLTV was introduced nationwide in Thailand during the reign of His Majesty King Bhumibol Adulyadej (King Rama IX) and was designed to bridge the knowledge gap for underprivileged children in remote rural areas (25). DLTV program is provided for an average of 4–5 h/day, 5 days/week. For each subject, there is 50 min of instruction for primary and lower secondary school students aired by the National Broadcasting and Telecommunications Commission (NBTC) networks. An additional 10 min is allocated for teachers to communicate directly with parents of DLTV students. Instruction for upper secondary school students is divided into a 30-min DLTV session and a 20-min pre-recorded video by a subject specialist. To facilitate two-way communication and to enhance understanding of the content, online discussion is arranged between teachers and students, depending on the availability of a device and Internet connection.

Apart from academic instruction, DLTV also airs various general interest programs (e.g., documentaries, news, environmental features, historical pieces, etc.) but tailored for a youth audience. Additional learning material (e.g., video documentaries, e-books) are also provided by teachers to enhance student learning. During relaxation of COVID-19 containment measures, teachers could conduct home visits to deliver and collect home-based learning materials or provide selective coaching to students and/or parents/guardians.

Just under one-fourth (23.6%) of this nationally representative sample of Thai students did not have a full access to a stable Internet and/or television signal at the time of the survey. These students were given handouts and written assignments to keep pace with the online learners (Figure 1). Every once or twice a week – depending on the agreement between the school and the families – the teachers sent the worksheets *via* post to students’ homes, and the family mailed back the completed assignments. However, for epidemic areas, this method burdened both teachers and families, particularly when the postal services were suspended or delayed due to severity of the COVID-19 outbreak. Thus, teachers had to use multiple strategies to ensure that the students received the worksheets and homework assignments. For example, some teachers used community radio or sent video clips *via* messaging applications (e.g., LINE or Facebook). Sometimes, teachers copied learning resources and assignments onto a USB flash drive, and distributed these to students *via* messenger service. In areas where COVID-19 containment measures had been relaxed, parents/guardians visited schools to collect the handouts from teachers and submit the completed assignments from the previous week.

Onsite (classroom) learning was implemented in some areas where COVID-19 had been declared “contained.” About 3.1% of Thai students (Figure 1) were able to resume schooling by September 2021 by adopting blended learning or hybrid methods (combination of onsite and other platforms). The field offices of the Ministry of Education coordinated with schools to organize staggered classroom learning to ensure that attendance did not violate social distancing guidelines. That way, students spent part of the week in the classroom and the rest of the week online. A variety of on-off strategies were applied by schools, such as the following: (1) Scheduling classes for alternating groups of students; (2) Scheduling attendance by odd- and even-number days of the month; (3) Staggering the schedule (i.e., 5 days onsite learning, 9 days home learning); and (4) Staggering attendance to the daily schedule (i.e., morning or afternoon classes).

To ensure the safety of students and teachers, a strict DMHTT regimen was practiced (Distancing, Mask wearing, Hand washing, Testing, and “Thai Chana”). For example, upon arrival at school, students washed their hands before entering the classroom. They had to wear a sanitary mask throughout the day. For students believed to be at risk of COVID-19, the school coordinated with the local clinical facility to conduct periodic screening. Students with “smart phones” (i.e., with Internet access) were requested to install the “Thai Chana” application for easy check-in/check-out from the places they visited as a monitoring measure. Although schools resumed in-person instruction, some limited classroom time to only several hours a day was implemented to reduce risk of infection.

### Correlation Between Learning Method During School Closures and Anxiety

Given the various learning methods being implemented during the school closure, about one in three (32.8%) Thai students experienced anxiety. Moderate-to-severe anxiety was most frequently reported among students who learnt fully (38.0%) or partially (29.9%) online. The proportion with some anxiety was lowest among students who studied in a traditional classroom (onsite) (Table 2). Bivariate analysis results confirm that, compared to other learning methods, students who had fully online learning were more vulnerable to experiencing anxiety (*F*-test 51.154; *p*-value <0.001).

**TABLE 2:** Cross-tabulation: learning method and anxiety level.

	Very mild-to-mild anxiety		Moderate-to-severe anxiety		
		
Learning method	N	%	N	%	Mean score
Fully online	2503	62.0	1532	38.0	2.05
Partially online	295	70.1	126	29.9	1.89
Handout/written assignments	1136	79.1	300	20.9	1.69
Onsite	152	81.7	34	18.3	1.59
Total	4086	67.2	1992	32.8	
Chi-square test	160.711***			
*F*-test	51.154***			

***Significant at p-value <0.001.

To further examine the correlation between learning method and anxiety, binary logistic regression analysis was employed with the covariates of sex, school grade, family and peer support, having a debilitating chronic disease, and area of residence. The results show that, when all other variables are held constant, onsite (i.e., in-person) learning had a significant inverse correlation with anxiety. Compared to fully online learning, students who attended a traditional classroom (onsite) were 37.8% less likely to report moderate-to-severe anxiety (OR 0.6; *p*-value 0.021). The likelihood of reporting moderate-to-severe anxiety was also lower among male students (OR 0.8; *p*-value <0.001), and younger students (primary and lower secondary school grades). Those who received adequate support from their families and peers were less likely to report anxiety. The probability of having moderate-to-severe anxiety was higher among students with a debilitating chronic disease (OR 1.4; *p*-value <0.001), residing in an urban area (OR 1.2; *p*-value 0.023), residing in the north region (OR 1.3; *p*-value 0.020), or residing in the central region (OR 1.3; *p*-value 0.022) (Table 3).

**TABLE 3:** Correlates of school closure-induced anxiety at a moderate-to-severe level.

	OR	*p*-value	95% C.I. for EXP(B)	
			
			Lower	Upper
**Learning method**				
**Fully online (Ref.)**				
Partially online	1.233	0.081	0.975	1.560
Handouts/written assignments	0.880	0.133	0.746	1.039
Onsite	0.622	0.021	0.416	0.930
**Sex**				
**Female (Ref.)**				
Male	0.792	0.000	0.707	0.888
**Education level**				
Lower primary	0.178	0.000	0.147	0.217
Upper primary	0.289	0.000	0.241	0.346
Lower secondary	0.627	0.000	0.525	0.749
**Upper secondary (Ref.)**				
**Family and peer support**				
**No (Ref.)**				
Yes	0.752	0.000	0.650	0.871
**Having a debilitating chronic disease**				
**No (Ref.)**				
Yes	1.439	0.000	1.207	1.716
**Place of residence**				
Urban	1.159	0.023	1.020	1.316
**Rural (Ref.)**				
**Region of residence**				
North	1.310	0.020	1.043	1.644
Northeast	0.907	0.416	0.717	1.147
Central	1.279	0.022	1.036	1.578
South	1.157	0.209	0.922	1.453
**Bangkok (Ref.)**				
Constant	1.441	0.010		
−2 Log likelihood		7026.837		
Cox and snell R square		0.103		
Nagelkerke R square		0.144		

### Correlation Between Learning Method During School Closures and Health Behavior

**FIGURE 2:**
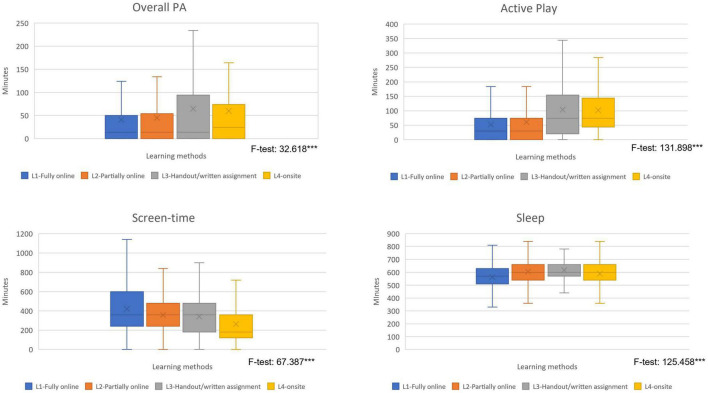
Average minutes of PA, active play, screen time, and sleep of Thai students during school closure. ***Significant at *p*-value <0.001.

Periodic school closure has also affected the health behavior of Thai students. With the transition from daily classroom instruction to various remote learning methods, youth experienced adverse changes to their daily routines. In the pre-COVID-19 period, Thai students accumulated an average of 53 min of MVPA (26). However, only 47 min of MVPA was recorded during the pandemic. Students who experienced a full- or partially online learning methods reported lower PA (41 and 45 min, respectively) than students who attended classroom learning or were taught by handouts and written assignments (65 and 60 min, respectively). Students who received handouts/written assignments or attended traditional classroom learning also had more opportunity for outdoor play (104 and 102 min, respectively) than their fully/partially online learning counterparts (53 and 61 min, respectively) (Figure 2). Correspondingly, the proportion of students who met PA and active play guidelines was significantly higher among those who learned by handouts/written assignments, or attended traditional classroom learning (Table 4).

**TABLE 4:** Cross-tabulation: learning method during pandemic-related school closures and health behavior.

Learning method		Overall PA		Active Play		Screen Time		Sleep	
					
		Meeting guidelines	Not meeting guidelines	Meeting guidelines	Not meeting guidelines	Meeting guidelines	Not meeting guidelines	Meeting guidelines	Not meeting guidelines
Fully online	n	959	3076	534	3501	506	3529	3255	780
	%	23.8%	76.2%	13.2%	86.8%	12.5%	87.5%	80.7%	19.3%
Partially online	n	103	318	73	348	64	357	370	51
	%	24.5%	75.5%	17.3%	82.7%	15.2%	84.8%	87.9%	12.1%
Handouts/assignments	n	526	910	489	947	269	1167	1296	140
	%	36.6%	63.4%	34.1%	65.9%	18.7%	81.3%	90.3%	9.7%
Onsite	n	49	137	63	123	72	114	158	28
	%	26.3%	73.7%	33.9%	66.1%	38.7%	61.3%	84.9%	15.1%
Total	n	1637	4441	1159	4919	911	5167	5079	999
	%	26.9%	73.1%	19.1%	80.9%	15.0%	85.0%	83.6%	16.4%
Chi-square		90.496***		325.150***		116.933***		161.184***	

***Significant at p-value <0.001. Meeting PA guideline: 60-min MVPA; meeting active play guidelines: being outdoors for play for more than 2 h a day; meeting screen time guidelines: not more than 2 h recreational screen time a day; sleep guidelines: 9–11 h for youth aged 6–13 years and 8–10 h for youth aged 14–17 years.

As expected, the shift from traditional classroom to distance/online learning required students to engage with screen media longer than recommended. In this nationally representative sample, Thai students accumulated an average of 393 min of recreational screen time per day (SD 235, CI 387-399), with the highest duration for fully online learning (421 min) or partially online learning (385 min) (Figure 2). The proportion of students who accumulated of more than 2 h recreational screen time was also the highest among those who were exposed to fully online or partially online learning methods (87.5 and 84.8%, respectively) (Table 4).

Learning method also had a significant correlation with sleep. The vast majority (more than 80%) of Thai primary and secondary school students met the recommended sleep duration guidelines, with the highest proportion of compliance among those who received handouts/written assignments (Table 4). Bivariate analysis employing the Chi-square test found that the association between learning method and health behavior was significant for all four observed variables.

### Correlation Between School Closure-Induced Anxiety and Health Behavior

This study found that school closure-induced anxiety significantly correlated with health behavior. The proportion of students meeting PA guidelines was higher (28%) among students who reported very mild-to-mild anxiety, compared to those who had moderate-to-severe anxiety (24.7%) during periods of school closure. Similarly, the proportion of students meeting active play guidelines was higher among those who reported very mild-to-mild anxiety (20.8% versus 15.6%). While the vast majority of students who reported anxiety at a moderate or severe level failed to meet the recommended screen time guidelines, most complied with the recommendations for sleep duration (Table 5).

**TABLE 5:** Cross-tabulation: anxiety and health behavior.

Anxiety		Overall PA		Active Play (AP)		Screen Time (ST)		Sleep (SL)	
					
		Meeting guidelines	Not meeting guidelines	Meeting guidelines	Not meeting guidelines	Meeting guidelines	Not meeting guidelines	Meeting guidelines	Not meeting guidelines
Very mild-to-mild	n	1144	2942	849	3237	722	3364	3566	520
	%	28.0%	72.0%	20.8%	79.2%	17.7%	82.3%	87.3%	12.7%
Moderate-to severe	n	493	1499	310	1682	189	1803	1513	479
	%	24.7%	75.3%	15.6%	84.4%	9.5%	90.5%	76.0%	24.0%
Total	n	1637	4441	1159	4919	911	5167	5079	999
	%	26.9%	73.1%	19.1%	80.9%	15.0%	85.0%	83.6%	16.4%
Chi-square		7.183**		23.608***		70.360***		124.934***	

***Significant at p-value <0.001, **Significant at p-value <0.01. Meeting PA guideline: 60-min MVPA; meeting active play guidelines: being outdoors for play for more than 2 h a day; meeting screen time guidelines: not more than 2 h recreational screen time a day; sleep guidelines: 9–11 h for youth aged 6–13 years and 8–10 h for youth aged 14–17 years.

The second set of binary logistic regression analysis was employed to investigate the correlation between school closure-induced anxiety and health behavior (PA, active play, screen time, and sleep). This study found there was no significant correlation between anxiety and overall PA or active play. The likelihood of meeting PA guidelines was determined by sex, learning method, family support, and residential characteristics. Similar to during the pre-pandemic period, male students were more likely to meet the 60-min MVPA daily threshold than their female counterparts (OR 1.5; *p*-value <0.001). In terms of learning modality, only handouts/written assignments had a significant correlation with PA. Compared to those who experienced fully online learning, students who received handouts/written assignments were more likely to meet the recommended PA guidelines. The significance of family and peers as a source of motivation is reflected by the higher odds of meeting PA guidelines among students who received adequate support (OR 2.2; *p*-value <0.001) (Table 6).

**TABLE 6:** Correlates of physical activity, screen time and sleep during pandemic-related school closure.

	Overall PA				Active Play				Screen Time				Sleep			
				
	OR	*p*-value	95% C.I.		OR	*p*-value	95% C.I.		OR	*p*-value	95% C.I.		OR	*p*-value	95% C.I.	
												
			Lower	Upper			Lower	Upper			Lower	Upper			Lower	Upper
**School closure-induced anxiety**																
**Very mild-to-mild (Ref.)**																
Moderate-to-severe	1.06	0.362	0.93	1.22	1.05	0.547	0.90	1.23	0.69	0.000	0.58	0.83	0.65	0.000	0.56	0.76
**Learning method**																
**Fully online (Ref.)**																
Partially online	0.98	0.869	0.76	1.26	1.24	0.135	0.93	1.65	0.84	0.259	0.63	1.13	1.08	0.624	0.78	1.50
Handouts/written assignments	1.49	0.000	1.28	1.74	2.10	0.000	1.78	2.48	0.87	0.132	0.73	1.04	1.12	0.305	0.90	1.40
Onsite	0.77	0.148	0.54	1.10	1.72	0.002	1.22	2.42	2.56	0.000	1.82	3.59	0.85	0.481	0.55	1.32
**Sex**																
**Female (Ref.)**																
Male	1.54	0.000	1.37	1.73	1.52	0.000	1.33	1.74	0.73	0.000	0.63	0.85	0.97	0.630	0.84	1.11
**Education level**																
Lower primary	1.18	0.138	0.95	1.46	1.80	0.000	1.38	2.33	4.23	0.000	3.07	5.83	5.63	0.000	4.36	7.27
Upper primary	1.01	0.925	0.82	1.24	1.28	0.064	0.99	1.65	2.58	0.000	1.87	3.55	2.32	0.000	1.88	2.86
Lower secondary	0.86	0.175	0.69	1.07	0.73	0.031	0.55	0.97	1.09	0.620	0.77	1.56	1.81	0.000	1.49	2.20
**Upper secondary (Ref.)**																
**Family and peer support**																
**No (Ref.)**																
Yes	2.17	0.000	1.80	2.62	1.67	0.000	1.34	2.09	1.23	0.069	0.98	1.53	1.33	0.001	1.12	1.58
**Having a debilitating chronic disease**																
**No (Ref.)**																
Yes	1.08	0.415	0.90	1.30	1.09	0.422	0.88	1.34	1.15	0.220	0.92	1.44	0.81	0.054	0.65	1.00
**Place of residence**																
Urban	0.77	0.000	0.67	0.87	0.68	0.000	0.59	0.78	0.92	0.296	0.79	1.08	1.01	0.859	0.86	1.19
**Rural (Ref.)**																
**Region of residence**																
North	0.94	0.618	0.74	1.19	1.62	0.006	1.15	2.28	1.11	0.515	0.81	1.53	1.25	0.111	0.95	1.66
Northeast	1.10	0.452	0.86	1.39	2.93	0.000	2.09	4.11	1.66	0.002	1.21	2.28	1.16	0.289	0.88	1.54
Central	0.46	0.000	0.37	0.59	1.30	0.121	0.93	1.81	1.32	0.066	0.98	1.77	1.23	0.113	0.95	1.58
South	1.04	0.725	0.83	1.32	2.01	0.000	1.44	2.82	0.98	0.915	0.71	1.35	1.02	0.868	0.78	1.34
**Bangkok (Ref.)**																
Constant	0.19	0.000			0.05	0.000			0.06	0.000			1.82	0.000		
−2 Log likelihood		6683.069				5342.895				4767.463				5029.088		
Cox and snell R square		0.06				0.09				0.06				0.06		
Nagelkerke R square		0.09				0.15				0.10				0.11		

Similarly, the likelihood of meeting active play guidelines was also significantly correlated with sex, learning method, family/peer support, and area of residence. Male students had a higher probability to engage in more than 2 h of outdoor play than female students (OR 1.5; *p*-value <0.001). Students who received handouts/written assignments or learned in a classroom setting were 2.1 and 1.7 times more likely to have an opportunity for active play, respectively. Similar to overall PA outcome, those who received support from their family and/or peers were more likely to meet the active play guidelines. By contrast, those who resided in an urban area were less likely to comply with the active-play recommendations (OR 0.7; *p*-value <0.001). While there was no correlation with overall PA, school grade was significantly correlated with active play. Compared to those in upper secondary school, students in lower primary and lower secondary school grades were more likely to accumulate more than 2 h active play in a day.

School closure-induced anxiety was significantly correlated with screen time and sleep. Students who reported moderate-to-severe anxiety were less likely to meet the recommended guideline for daily hours of screen time (OR 0.7; *p*-value <0.001) and sleep (0.6; *p*-value <0.001). Sex was correlated with meeting the screen time guidelines, but had no significant correlation with sleep duration. Likewise, learning method was only correlated with screen time compliance; students who attended a traditional classroom met the recommended limit of 2 h recreational screen time daily (OR 2.6; *p*-value <0.001). School grade was correlated with both screen time and sleep duration; primary and lower secondary school students were more likely to meet the guidelines than upper secondary school students. Family/peer support was only correlated with sleep duration compliance, whereas place of residence (urban/rural) showed no significant correlation with either screen time or sleep. Region of residence, however, was significantly correlated with screen time; students who resided in the northeast region were 1.7 times more likely to meet the recommended guidelines.

## Discussion

### Correlation Between Learning Method and School Closure-Induced Anxiety

As a setting where youth spend a considerable amount of their time, schools play an important role in promoting health behavior among students. Besides developing cognitive skills, schools also serve as an ideal environment where children acquire lifelong social and emotional skills from interacting with their peers (6). The advent of COVID-19, therefore, has undoubtedly disrupted some of the developmental milestones for affected youth, and adversely impacted on health behavior. The closure of schools has forced students to be at their home or the immediate neighborhood for nearly 2 years (as of November 2021), limiting their outdoor activities, and isolating them from their peers (10, 27).

This study found that about one in three Thai primary/secondary school students in Thailand experienced a school closure-induced anxiety of a moderate-to-severe level. School closure-induced anxiety was reported more frequently among students who were taught by fully or partially online methods. The shift from traditional classroom to distance learning has pressured youth to make a significant adjustment to their formal education as well as their daily routine. Although adolescents are perhaps more adept than other age groups with digital technology (28), the changes in the learning environment may have adversely affected their ability to absorb the academic content in a timely way and, thus, lead to anxiety. Online learning limits the opportunity to have a thorough discussion with teachers and peers (3), particularly on the subjects that require practice or calculation (e.g., math, physics). Having to learn through online screen time for 5–6 h a day undoubtedly led some students to experience physical and visual fatigue. That strain can worsen into clinical anxiety. Some students may also experience anxiety from the increased homework during the COVID-19 era (10, 29). The social distancing and travel restrictions severely reduce the opportunity for peer-based leisure interaction. Connectedness with one’s peers has been reported in many studies as one of the key factors behind a student’s overall satisfaction toward different learning formats (8, 29).

Male and younger students reported lower level of anxiety during periods of school closure. This result corresponds with several studies on meta-cognitive belief that female students have a higher degree of perceived uncontrollability compared to their male counterparts (30–32). More than males, female students are more likely to express their anxiety and worries with regard to a school closure and its impact on their academic performance. By contrast, male students may be expected to exhibit a sense of control over an adverse situation or remain reticent.

Similar to male students generally, younger students are more “laid-back” in reacting to school closures. The lower likelihood of younger students to experience moderate-to-severe anxiety could also be seen as an effect of the learning format; 40.7% of the younger (early primary) students received handouts/written assignments, whereas the vast majority (more than 90%) of older (secondary) students studied only online. The handout/assignment method offers a higher degree of flexibility in managing a student’s learning schedule compared to other methods. While some families relied heavily on the traditional classroom schedule to structure the child’s learning during the school closure, working parents/guardians may have enjoyed the increased flexibility of home learning and managed the learning schedule at their convenience. This method is similar to home schooling in general, where parents serve as a one-on-one tutor, and can observe their child as they learn. Therefore, the family plays an important role in creating a regular routine for studying, arranging a quiet space and learning environment, and coaching the child so that the student understands and can complete the lessons on time. Indeed, some younger students may have enjoyed home learning with family support more when compared to the experience of the older students who were required to study alone and entirely online. Although the relationship between handout/assignment learning and anxiety was not significant in the multivariate analysis, the path analysis showed a significant and direct negative association between the two variables (Supplementary Material).

### Correlation Between Learning Method and School Closure to Health Behavior

Previous studies on how school closure has affected mental health of children have been documented widely. Amid the COVID-19 pandemic-related measures globally, one out of four school-age youth are exhibiting anxiety or depressive symptoms (10, 33, 34). Anxiety in this study, however, refers to “school closure-induced anxiety” that is manifested in a student’s feeling or reaction to a school closure. Therefore, anxiety in this study should not be seen as the mental health outcome, but rather as an intermediate variable that may have an association with youth health behavior later on.

**FIGURE 3:**
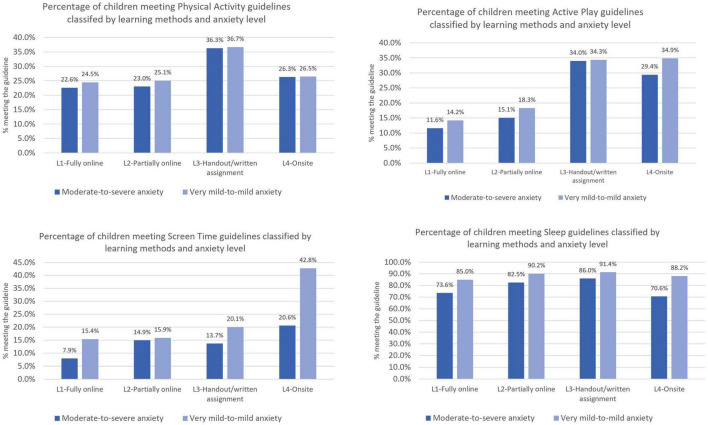
Adjusted proportional percentage of meeting health behavior guidelines by learning method and school closure-induced anxiety.

This study expected to see a correlation between school closure-induced anxiety and PA, active play, screen time, and sleep during the Thai response to the COVID-19 pandemic. However, anxiety was only significantly correlated with screen time and sleep duration. Although the bivariate analysis captured the association, binary logistic regression showed that there was no significant correlation between school closure-induced anxiety and overall PA or active play. Path analysis was applied to examine the direction between anxiety and health behavior. The results indicate that the direct and indirect association between school closure-induced anxiety with PA/active play was not significant (Supplementary Material). However, further investigation on the combined effect of school closure-induced anxiety and learning method show that the proportion of students who met the threshold of 60 min MVPA daily was the highest among those who received handouts/written assignments and experienced very mild anxiety. The proportion of students who engaged in 2 h or more of active play outdoors per day was the highest among students who attended traditional classroom learning with very mild level of anxiety (Figure 3).

The results of the binary logistic regression (Table 6) show that only the handouts/written assignments method has a significant correlation with PA, but the two methods (handouts, onsite/traditional classroom) significantly correlated with active play. It can be understood from the nature of handout/assignment method that handed parents and children a full authority in managing their learning schedule that also provided more opportunities for PA and outdoor play. The traditional classroom format did not have a significant effect on PA because of the lack of flexibility in the nationwide compulsory education curriculum. Even after in-school learning resumed in Thailand (late 2021) most structured PA programs (e.g., organized sport, PE classes) remained suspended or reduced.

The results of this study also imply that free play is a natural form of PA for children. Thus, the two learning methods (i.e., handouts/written assignment and onsite) may correlate more with active play. For children, active play is usually something that is done with peers or siblings. In that sense, the handouts/written assignments and traditional classroom are the two formats that would best accommodate the child’s need for play. Even though students who are home-schooled alone lose the opportunity to interact with classmates, they may have more free time to play with the extended family members and neighbors. Students who are able to attend school have more opportunity for playing outdoors with their classmates during recess and before/after school. A school’s policy in managing blended learning (i.e., 5 days onsite, 9 days online, or odd-even days) also may have provided additional chances for students to engage in PA and outdoor play.

The findings from this study suggest that the fully and partially online methods did not provide adequate opportunity for students to engage in PA or to play outdoors. Although a statistically significant relationship could not be established by binary logistic regression analysis, the *F*-test (Figure 2) shows that there was a significant difference in the cumulative minutes of PA and active play across learning formats. Students who studied full-time/partially online collected fewer minutes of PA than those who studied by handouts/written assignments or attended a traditional classroom. The highly structured nature of fully online learning (>5 h/day for 5 days/week) with additional assignments and homework greatly limit the student’s free time for PA and outdoor play.

When learning method was tabulated by school grade, (data not shown) this study found that nearly all (95.0%) of the secondary and more than half (57.1%) of late primary school students engaged in fully online learning. In today’s digital world, as a child ages, they tend to become more familiar and avid users of online technology. Thus, the older students probably had the least difficult time in shifting from classroom to online learning formats. However, studies have found that online education also serves as a bridge to a more virtual social environment (e.g., gaming and social media) which may have little or no academic benefit. The more time spent in the virtual world also deprives youth of engaging in nature and natural settings with others (i.e., PA, active play) (35).

School closure-induced anxiety was significantly correlated with screen time. Students who were displeased by school closures were more likely to engage in recreational screen time more in excess of the recommended threshold. A bulk of evidence has reported an increase of screen time among school-age youth worldwide, ranging from 0.5 to 5.9 h a day during the pandemic (14, 36). Older youth use online social media as an escape from the boredom of home or neighborhood confinement. By contrast, younger children spend more time watching television and hand-held gaming devices (35, 37, 38).

Adherence to recommended limits of screen time was also significantly associated with learning method. The binary logistic regression analysis (Table 6) suggests that only students who attended school onsite were more likely to comply the recommendation of less than 2 h recreational screen time per day. The combined effects of school closure-induced anxiety and learning method also show that the proportion of students who comply with the screen time limits was highest among those who attended school in person, who also reported the least anxiety (Figure 2). These findings suggest that traditional classroom attendance prevents youth from experiencing school closure-induced anxiety (at a moderate-to-severe level) because their need for free play was fulfilled from being out of home confinement and having peer interaction at school. That said, during the period of offsite learning, younger students enjoyed a considerable amount of play time with their siblings/extended family at home or in the immediate neighborhood. What is more, students who perceived their learning as enjoyable accumulated less screen time.

School closure-induced anxiety was not only correlated with student anxiety; it was also significantly correlated with sleep. The results of the binary logistic regression suggest that youth who reported school closure-induced anxiety at a moderate-to-severe level were less likely to meet the sleep duration guidelines. The path analysis confirmed the direct negative association between the two variables (Supplementary Material). It should be noted, however, that not meeting the sleep guidelines could be manifested in a longer or shorter duration of sleep than is recommended. Because the TRC design is a cross-sectional survey, this study could not document the changes in the sleeping pattern during the pandemic. Previous studies have investigated these patterns, and found that, in the absence of school routines and less exposure to the outdoor environment, children experienced a longer duration of sleep (6, 10). On the other hand, those who were more adversely affected by school closures may be prone to a higher devotion to screen time, resulting in sleep deprivation (12, 35).

Although the correlation between learning method and sleep could not be established from the binary logistic regression, path analysis showed a significant, positive, direct association between L2 (partially online study), and L3 (handouts/written assignments) and sleep compliance (Supplementary Material). Further, instead of the learning method, school grade (level) played a significant role in determining sleeping patterns. Younger students were more likely (OR 5.6; *p*-value <0.001) to meet the recommended sleeping guidelines, compared to their older counterparts (Table 6). This finding points to the significance of family roles in regulating a younger child’s behavior during the pandemic, though that may not be the case for adolescents. The analysis of the correlation of learning method on anxiety (Figure 3) also shows that the proportion of students who complied with sleeping guidelines was highest among those who attended a traditional classroom, and who also had the least anxiety. Again, traditional classroom attendance in this study was mostly filled by younger students because the vast majority of secondary school students (i.e., adolescents) shifted to online learning. Studies have established that adolescence is the period when children begin to seek exogenous resources (e.g., peers instead of their parents) for their experiences, companionship, and approval (39). Parental roles, therefore, become less significant for adolescents, compared to younger children.

This study has provided important evidence in establishing the correlation between various learning methods implemented in Thai primary and secondary school during the government’s response to the threat of COVID-19 with health behavior of students. As the sample of this study is nationally representative, the results should portray the current learning situation in Thailand with regard to pandemic-related school closures and student anxiety. The study has also rigorously demonstrated the effect of various learning methods on school closure-induced anxiety and health behavior (PA, active play, screen time, and sleep). Nevertheless, a few limitations of this study should be acknowledged. First, because of the cross-sectional nature of the TRC survey (which is the source of the data), causal relationships between variables could not be statistically determined. Future research, therefore, should employ a longitudinal design (i.e., panel data) to better observe the effect of learning methods to students’ anxiety and health behaviors. Secondly, the level of school closure-induced anxiety was assessed from a single question and, thus, is perhaps less sensitive for younger children. However, the use of a flashcard was considered appropriate to objectively assess a child’s emotional response. Third, subjective assessment on PA, screen time and sleep could lead to recall bias. Future research, therefore, should employ objective measures to determine PA level, screen time, and sleep duration.

## Conclusion

Online learning may be seen as an innovative approach in education and is considered as the most feasible method for delivery of formal education during a prolonged national crisis. However, this study found that the shift to online learning is also significantly correlated with anxiety and youth health behavior. School closure-induced anxiety at a moderate-to-severe level was prevalent among students who were required to engage in fully or partially online instruction methods. By contrast, anxiety was less prevalent among students who attended a traditional classroom for in-person learning. A higher use of screen media combined with additional homework and written assignments have burdened Thai students and limited their opportunity to engage in healthy behavior (PA or active play).

The offline learning methods (handouts, onsite) were significantly correlated with a higher probability of students to meet all four health behavior guidelines (PA, active play, screen time, and sleep) and be less prone to severe anxiety. The significance of handouts/written assignments in facilitating PA, and the two learning formats (handouts, classroom) for active play suggest that unstructured play is a natural form of PA for children. These two instructional formats provide adequate opportunity for children to engage in PA, either at home with their siblings or at school with their classmates. The findings of the study also suggest that parents/family play a significant role in regulating a child’s schedule during home schooling.

The results of this study should be beneficial for Thai government and policy makers in evaluating learning models for primary and secondary schools to prevent further learning loss during a pandemic. Although the spread of COVID-19 in Thailand has tended to subside (as of late 2021) and schools are now preparing for reopening, there is still a possibility of a resurgent epidemic of new strains of COVID-19 or other pathogens in the near future. The government and schools, therefore, should be prepared in considering future educational strategies during a prolonged national crisis. If online learning remains the most applicable option, the new model should reduce the duration of learning on-screen and provide more opportunity for active learning in the home setting to prevent debilitating anxiety. As parents/guardians can play a significant role in their child’s education, communication between schools and families of students should be enhanced in order to improve the design of learning programs and constructive activities to meet the desired educational and health outcomes.

## Data Availability Statement

The original contributions presented in the study are included in the article/Supplementary Material, further inquiries can be directed to the corresponding author.

## Ethics Statement

The studies involving human participants were reviewed and approved by Institutional Review Board of the Institute for Population and Social Research, Mahidol University (COA. No. 2021/05-115). Written informed consent to participate in this study was provided by the participants’ legal guardian/next of kin.

## Author Contributions

DAW, PK, PS, and NR conceptualized the study. DAW, PK, and SR conceived the study. DAW and PK drafted the manuscript and interpreted the results and wrote the final manuscript. PK performed the formal data analysis. All authors reviewed and contributed to the final manuscript and read and approved the final version of the manuscript, and agreed with the order of presentation of the authors.

## Conflict of Interest

The authors declare that the research was conducted in the absence of any commercial or financial relationships that could be construed as a potential conflict of interest.

## Publisher’s Note

All claims expressed in this article are solely those of the authors and do not necessarily represent those of their affiliated organizations, or those of the publisher, the editors and the reviewers. Any product that may be evaluated in this article, or claim that may be made by its manufacturer, is not guaranteed or endorsed by the publisher.
