# Supplementary material for: Learning Methods During School Closure and Its Correlation With Anxiety and Health Behavior of Thai Students
Source: Front Pediatr. 2022 Mar 28;10:815148. doi: 10.3389/fped.2022.815148 (PMC8995846; doi:10.3389/fped.2022.815148)
Supplement: Supplementary file 1 [file Data_Sheet_1.PDF]

Supplement 1. Path analysis on the correlation between learning methods, anxiety and healthy behaviors

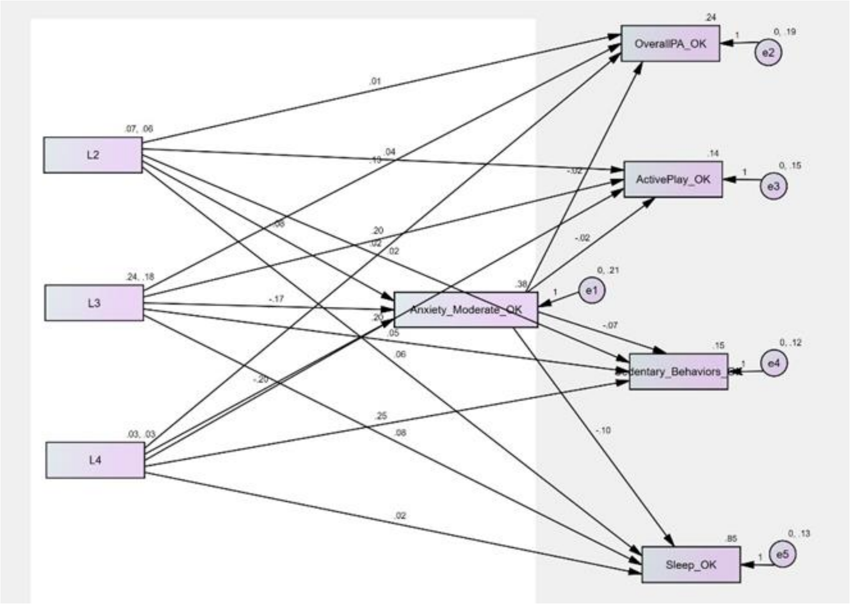

Regression Weights: (Group number 1 - Default model)

			Estimate	S.E.	C.R.	P	Label
Anxiety_Moderate_OK	<---	L2	-.080	.024	-3.389	***	par_1
Anxiety_Moderate_OK	<---	L3	-.171	.014	-11.998	***	par_2
Anxiety_Moderate_OK	<---	L4	-.197	.035	-5.668	***	par_3
OverallPA_OK	<---	L2	.006	.023	.253	.800	par_4
ActivePlay_OK	<---	L2	.039	.020	2.007	.045	par_5
Sedentary_Behaviors_OK	<---	L2	.021	.018	1.162	.245	par_6
Sleep_OK	<---	L2	.064	.019	3.416	***	par_7
OverallPA_OK	<---	L3	.126	.014	9.198	***	par_8
ActivePlay_OK	<---	L3	.204	.012	17.214	***	par_9
Sedentary_Behaviors_OK	<---	L3	.050	.011	4.561	***	par_10
Sleep_OK	<---	L3	.078	.011	6.902	***	par_11
OverallPA_OK	<---	L4	.023	.033	.684	.494	par_12
ActivePlay_OK	<---	L4	.202	.029	7.034	***	par_13
Sedentary_Behaviors_OK	<---	L4	.248	.026	9.364	***	par_14
Sleep_OK	<---	L4	.023	.027	.825	.409	par_15
OverallPA_OK	<---	Anxiety_Moderate_OK	-.016	.012	-1.307	.191	par_16
ActivePlay_OK	<---	Anxiety_Moderate_OK	-.022	.011	-2.049	.040	par_17
Sedentary_Behaviors_OK	<---	Anxiety_Moderate_OK	-.070	.010	-7.209	***	par_18
Sleep_OK	<---	Anxiety_Moderate_OK	-.102	.010	-10.103	***	par_19
